# Positive effects of the COVID-19 pandemic on depression and anxiety in Chinese adolescents

**DOI:** 10.1007/s00787-023-02263-z

**Published:** 2023-07-24

**Authors:** Xinhua Yang, Andrew J. Lawrence, Phillippa Harrison, Yanlong Liu, Liangliang Chen, Chenglei Wang, Chao Yan, Roland Zahn

**Affiliations:** 1 Changning Mental Health Center, 299 Xiehe Road, Changning District, Shanghai, 200335 China; 2 Centre for Affective Disorders, Psychological Medicine, Institute of Psychiatry, Psychology and Neuroscience, King’s College London, London, UK; 3 The Affiliated Kangning Hospital, Wenzhou Medical University, Wenzhou, China; 4 School of Psychology and Cognitive Science, East China Normal University, Shanghai, China; 5 South London and Maudsley NHS Foundation Trust, London, UK

**Keywords:** Depression, Anxiety, COVID-19, Lockdown, Adolescence

## Abstract

The potential impact on mental health of home schooling and social isolation due to COVID-19 lockdowns has led to widespread concern, particularly for adolescents. However, studies including pre-pandemic data from longitudinal cohorts with an assessment of the longer-term impact of the Covid-19 pandemic beyond the first months of 2020 are scarce. This longitudinal study of 1534 adolescents attending a secondary school in Hunan province investigated self-reported symptoms of anxiety and depression using two validated scales (Screen for Child Anxiety Related Disorders, Child Mood and Feelings Questionnaire) at six time points before, during, and after the 2020 national lockdown restrictions in China. Perceived COVID-related stress was assessed by an author-developed scale at two timepoints during the lockdown. We investigated trends in symptoms over time with a fixed effects model and multiple imputations of missing data. Counter to our expectations, depressive and anxiety symptoms were reduced during the 2020 lockdown relative to pre-lockdown (depression: *b* = − 3.37, SE = 0.345, Cohen’s *d* = − 0.25, *p* < 0.0001; anxiety: *b* = − 4.55, SE = 0.382, Cohen’s *d* = − 0.30, *p* < 0.0001). Symptoms remained significantly reduced even after lockdown restrictions eased. Higher symptom levels during lockdown were associated with greater self-reported COVID-related stress (depression: *b* = 0.11, SE = 0.026, *p* < 0.0001; anxiety: *b* = 0.11, SE = 0.036, *p* < 0.0001). Although COVID-related stresses correlated with higher levels of anxiety and depression, the lockdown period was associated with improved symptom levels in the adolescents taking part in our study. School closures may have improved the mental health of adolescents in China. We speculate this beneficial effect of lockdown can be explained by the adverse effects of attending school itself such as exposure to bullying and achievement pressures.

**Supplementary Information:**

The online version contains supplementary material available at 10.1007/s00787-023-02263-z.

## Introduction

The COVID-19 pandemic continues to raise profound and long-lasting concerns about the population's mental health, particularly in adolescents. In China, following the first national lockdown in the spring of 2020, subsequent control measures were local in scope with the implementation of the “Dynamic COVID-zero” strategy. This contrasts with the recurring national restrictions adopted in many Western countries. However, the prevention and control measures including local strict quarantine, travel and border control, large-scale PCR testing, and decreased human mobility. The chronicity and comprehensiveness of the lockdown restrictions may have caused an upsurge in anxiety, complaints, frustrating and mental health problems under severe uncertainties. This could be particularly problematic for children and adolescents as they may have less psychological and social resources to handle chronic stress. A meta-analysis study based on cross-sectional design revealed increased levels of mental health problems in among Chinese children and adolescents during the lockdown [1]. For other countries, several systematic reviews of the prevalence of depression and anxiety also showed a significant increase in the initial phase of the COVID-19 outbreak [2, 3]. However, these studies lack pre-lockdown information which makes it difficult to characterise the time course of the mental health burden associated with the pandemic. Furthermore, longitudinal studies on adolescent psychological profiles covering the entire period of the pandemic from pre- to post-lockdown are lacking.

Contrary to concerns raised early in the pandemic, longitudinal studies on adolescents’ mental health, including ours [4], reported that lockdown had minimal or no impact on mental health compared to pre-pandemic level [5, 6], even an improvement in wellbeing [6, 7]. Another recent study reviewed 65 longitudinal cohort studies including adults/children/adolescents comparing mental health prior to and during a pandemic and found a small increase in depression in the early stages of the pandemic and then a decrease over the following months with a period of psychological adaptation and resilience [8]. Similar findings were also found in the general population: a review based on 25 longitudinal studies concluded that the psychological impact of the Covid-19 lockdowns was small on average compared with pre-lockdown levels, and suggested that most people were resilient during the first months of the pandemic [1]. Notably, several large-scale studies that included pre-pandemic data did not find evidence of a worsening of mental health symptoms among samples of patients with pre-existing mental health conditions [9, 10]. Adolescents with high pre-pandemic symptoms reported no change in emotional symptoms [8, 11] or a decrease in both internalizing problems and externalizing problems during the pandemic [12]. These reports are consistent with growing evidence that some people exposed to acute adversity see improved mental health functioning from before to after exposure or psychosocial gains from adversity [13].

One explanation for these improvements is that acute stress and pandemics could stimulate family and social connectedness, trustworthiness and sharing behaviour, prompting a greater sense of solidarity and bonding [14, 15]. Indeed, the pandemic’s effects on mental health will be heterogeneous across individuals, situations and contexts [13]. Although the risk of harm to mental health is considerable, a number of protective factors may mitigate these harms. As research has shown, people have the capacity to successfully adapt and even flourish following large-scale stressful life events [16]. The pattern of adaptive functioning, or resilience, is likely to be shaped by distinct individual characteristics, but also by the socio-political context of lockdown restrictions [17]. Cultural beliefs about adversity can also influence how people adjust to COVID-19 [18]. In Confucian philosophy, adversity is an opportunity for the self-cultivation of virtues, and Chinese people may have strong forbearance and perseverance in the face of severe limitations on the capacity for in-person social interaction which might differ from other cultures [19, 20]. This highlights the need to consider culture-specific factors when investigating and interpreting the mental health effects of the pandemic.

For China, there were few longitudinal studies that have been able to map out how adolescents’ mental health has responded over the longer term, after the initial reaction to the Covid-19 pandemic relative to pre-Covid levels. Therefore, the present study aimed to address this important gap in evidence by investigating the temporal evolution of depressive and anxiety symptoms over the course of the pandemic. We had the unique opportunity of studying a sample of adolescents attending secondary school with available pre-pandemic data from an ongoing study. Here we investigated the following research questions:

1. Did symptoms of anxiety and depression worsen or improve during the initial pandemic lockdown?
2. Did these changes in symptoms persist or return to pre-COVID levels in the period post-lockdown?
3. Was the experience of pandemic-related stress associated with changes in symptoms of anxiety and depression in the initial lockdown.

## Methods

### Participants

Participants in Years 10–11 were recruited from a single secondary school in Chenzhou city (Hunan Province, China) as a part of an ongoing (October 2018-present) longitudinal study on bullying, victimization, and mental health among adolescents. In the Chinese education system, Year 10 (ages 15–16) is the first of three years of high school or "senior secondary" education, the last being Year 12 (ages 17–18) which is followed by university in those continuing academic education. The mental health questionnaires used in this study are completed and recorded routinely for non-research purposes (to monitor student mental health) and were only used for research purposes with informed consent. Data from six time points (see below) are included, labelled Time 1 to 6 and henceforth abbreviated: T1–T6. At the commencement of the study (T1, October 2018) and again in April 2020 (T4) students were invited to participate in the study and by doing so have their routinely collected scores used for research purposes. In total 1557 students provided informed consent to participate in the study, and, of these, 1534 (98.5%) could be linked to completed questionnaire data acquired from one or more study time points. Participants were predominantly female (*n* = 1060, 69.1%) and mostly aged 15–16 at the start of the study (age 14: 7.2%, 15: 61.2%, 16: 27.6%, 17: 3.5%, 18: 0.5%).

The 23 subjects who consented but could not be identified in the records were not significantly different from the rest of the sample in terms of sex (*p* = 0.07, Fisher’s exact test) or mean age (*p* = 0.887, *t* test).

**Fig. 1:**
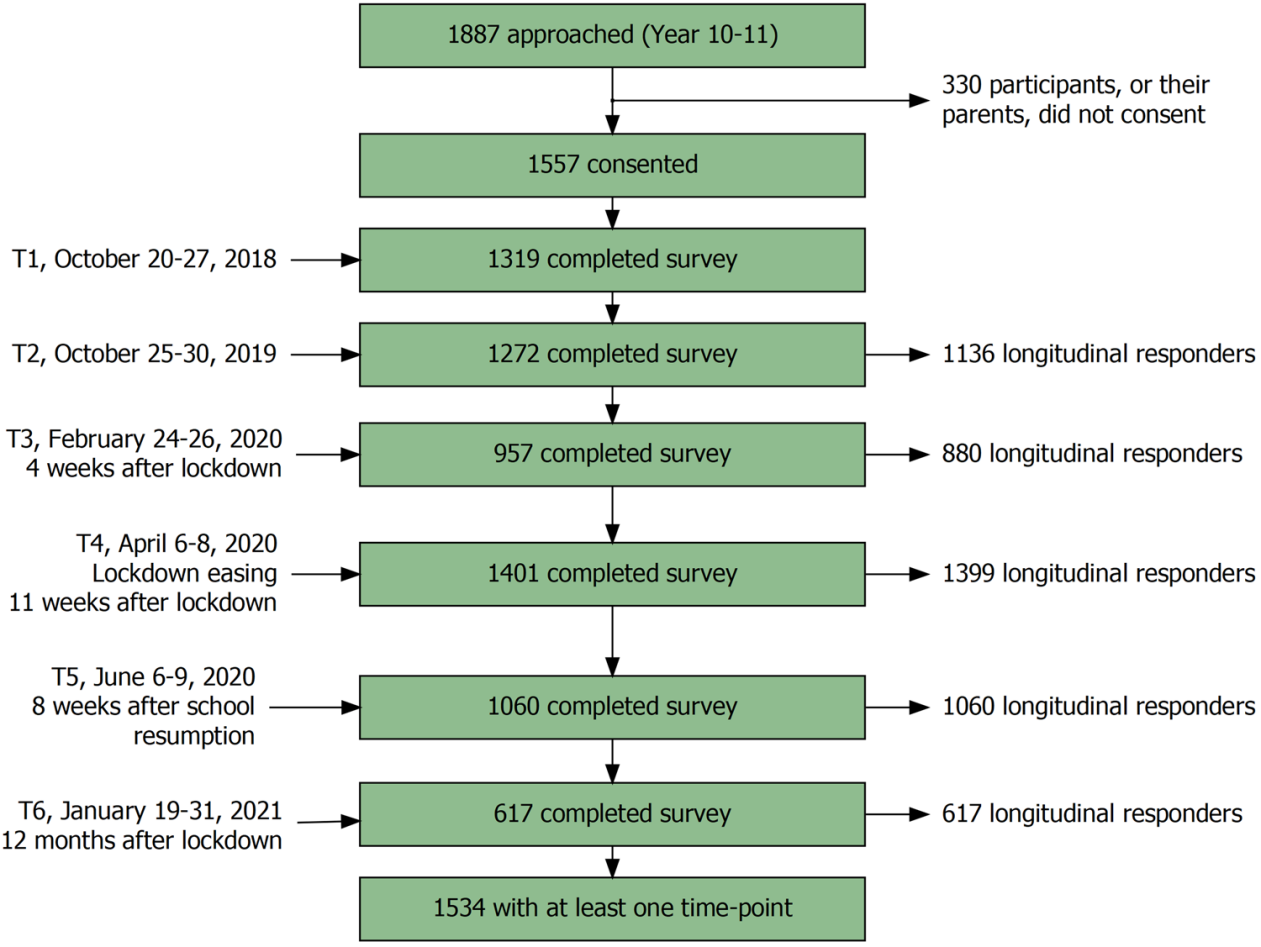
Flow chart of participants included in the study. A study flow chart with the number of participants who were approached, consented, and provided data at each time point of the study. There were 1506 participants with ≥ 2-time points; 1419 with ≥ 3-time points; 1119 with ≥ 4-time points; 769 with ≥ 5-time points; and 279 with all 6-time points. 23 consented participants could not be matched to records and did not contribute to analyses

No participants were excluded, but records that could not be linked to a participant who provided informed consent were not analysed (*n* = 1207, 14.7% of records; T1 = 58, T2 = 95, T3 = 146, T4 = 445, T5 = 261, T6 = 202). Further to this, repeat online submissions (i.e. T3 and onwards) that were logged from a subject ID who had already completed a submission for that survey were excluded (328 records total; T3 = 12, 1.2%; T4 = 117, 7.7%; T5 = 124, 10.5%; T6 = 75, 10.8%). Figure 1 presents a flowchart of participants with records identified at each time point.

### Ethical considerations

Informed consent was obtained from all individual participants and their parents or guardian. Participation was voluntary and no incentive or reward was given. Anonymity was emphasized; however, participants were asked to provide Pinyin abbreviation of their name to match across timepoint. It took participants approximately 30 min to complete the survey. The study protocol was approved by the Wenzhou Medical University ethics committee (2020–131).

### Timepoints

Mental health scores were obtained twice prior to COVID: T1 in October 20–23, 2018, n = 1319 records (68.9% female; Mean age = 15.29, SD age = 0.68); and one year after, T2: October 25–30, 2019, n = 1272 records (69.7% female; Mean T1 age = 15.27, SD = 0.68).

The third survey (T3, *n* = 957, 73.2% female; Mean T1 age = 15.31, SD = 0.67) was conducted between February 24–26, 2020, four weeks after the start of Chinese lockdown, during which time China was going through a phase of rapid increase in the number of COVID-19 cases and associated deaths. At this point, students had not attended school for six weeks (because the lockdown was timed just after the Chinese New Year national holiday). Participants were again invited to complete the COVID-19 effect on mental health survey with invitations were sent to students and their parents via a family-school communication app by the school mental health service.

The fourth survey (T4, *n* = 1401, 69.0% female; Mean T1 age = 15.25, SD = 0.64) was conducted from April 6 to 8, 2020, 11 weeks after the start of the lockdown. The Chinese government announced a step-by-step easing of the lockdown schedule, after evidence of a rapid decline in the number of new and suspected cases of COVID-19. Students had not attended school for 13 weeks at this time point.

The fifth survey (T5, *n* = 1060, 74.0% female; Mean T1 age = 15.20, SD = 0.61) was conducted from June 6 to 9, 2020, when there was a low number of cases, most social activities had resumed as normal, and students had been back at school for eight weeks (resumed school on April 13, 2020).

The sixth survey (T6, *n* = 617, 68.6% female; Mean T1 age = 15.08, SD = 0.54) was conducted from January 19 to 31 2021, around one year after the start of lockdown. Social and school life were normal, but Covidpass was necessary to access public or transportation, and smaller-scale local lockdowns were common under the "dynamic zero" policy.

### Outcomes and procedure

#### COVID 2019 stressful events scale

This self-rated scale was developed by the author (X.Y.) to evaluate stress experienced due to the COVID 2019 pandemic [21]. The following 16 items were included as potential causes of stress: (1) restrictions on the free movement of citizens, (2) testing of temperature and wearing masks in public, (3) closed residential community, (4) being unable to meet friends and relatives, (5) widespread news and information about the new virus, (6) daily reporting of their health situation, (7) being unable to exercise outdoors, (8) school closures, (9) learning online rather than face-to-face, (10) parents’ management of children’s learning rather than teachers, (11) family’s fears of COVID-19, (12) healthcare staffs infection and risk of hospitals being overwhelmed, (13) lack of supplies of personal protective equipment (PPE) i.e. masks or protective clothes, (14) confirmed cases in your area, (15) increasing daily incidence, (16) increasing daily deaths toll. Participant were instructed to report how stressful they found the influence of these items from 1 (not at all) to 5 (very severe). For analysis, we use a total score, which sums all individual item scores to reflect both the number and intensity of stressful experiences related to COVID/lockdown. The internal reliability (Cronbach’s Alpha) of this scale was “excellent” > 0.9 (0.93 at T3 and 0.95 at T4).

#### Anxiety symptoms

The Chinese version of the Screen for Child Anxiety-Related Emotional Disorders (SCARED) was used to screen for signs of anxiety disorders in children [22]. The SCARED is a 41-item inventory developed for ages 9–18 with five factors: generalized anxiety, separation anxiety, social phobia, school phobia and panic/somatic anxiety. Items were rated on a 3-point Likert scale: 0 (not true) to 2 (very true), with lower total scores indicating a higher risk of anxiety disorders.

#### Depressive symptoms

The Chinese version of the Child Mood and Feelings Questionnaire (MFQ-C) was used to measure self-reported experiences of depression [23]. The MFQ-C comprises 33 items rated on a 3-point Likert scale: 0 (not true) to 2 (true), with lower scores indicating greater depressive symptom severity. The MFQ-C was developed and validated for ages 6–19.

#### Procedure

The study was part of a school mental health program, implemented in line with Chinese guidelines to improve mental health in primary and secondary schools (2012) 15). Data collection at T1 and T2 took place via a paper-and-pencil survey during a regularly scheduled class period in the school setting. During the national lockdown, and after (T3–T6), data collection was conducted through an online system called ‘Wen Juan Xing’, distributed on Wechat social media platforms. The COVID-19 stressful events questionnaire was administered only at T3 and T4.

### Statistical analysis

Statistical analyses were carried out in three phases. The first phase describes the key COVID-19-related stressors reported by the sample. Next, a fixed effects approach to panel data was used to assess the secular effects of lockdown/COVID on depression and anxiety. In the third phase, COVID-19 stress total score was added to the models from the second phase to probe any impact on lockdown/COVID-related changes in depression and anxiety symptoms. For models in phase 3, we additionally include gender, and age at baseline, in the model and consider whether these covariates have a statistically significant impact on the effects of interest.

All tests are two-tailed with an alpha threshold of 0.05 for statistical significance. Multiple comparison corrections use the Holm step-down procedure [24].

An investigation of longitudinal measurement invariance for the MFQ and SCARED scales was carried out and identified no significant problems (see supplementary materials).

### Missing data

There was no missing data for age and gender which were acquired during the consent process, however, sporadically missing questionnaire data from one or more time points was common. A total of 6626 of a possible 9204 records (72%) were matched over the 1534 participants and 6 time points. For the key comparison of pre-lockdown vs. post-lockdown, 1420 (92.6%) participants had at least one observation of each type and so could contribute information to effect estimates. Of the remaining 114 subjects, 35 could be linked to only pre-COVID data and 79 to only post-COVID data.

To address missing data in the outcome variables and the COVID-19-related stressors questionnaire, we used multiple imputations—particularly the chained equations method in the R package “mice” [25]. MFQ and SCARED total scores over all six-time points were imputed along with the COVID stressful events questionnaire total score (acquired just at T3 and T4). Age at baseline and gender were included as auxiliary variables. Fifty iterations of Predictive Mean Matching were employed and 100 imputed datasets were generated. This is an “FCS-Standard” approach (based on the taxonomy in a recent overview of imputation methods for longitudinal data [26]. This is appropriate as we have homogenous timing of assessments and a small number of longitudinal observations/variables relative to the sample size. This approach is unstructured with respect to timepoint and does not assume the parameterisation of time employed in the linear mixed models described above. There were no convergence problems, confirmed by visual inspection for lack of trend in the line plots of each variable’s mean and variance during imputation plotted against iteration number [27]. Note that the covid stressful events individual items were not imputed, only the total score, as a result the item-level descriptive analysis excludes missing observations.

Results for multiply imputed data were pooled using Rubin’s rules [28], with small sample correction for degrees of freedom [29]. Coefficient tests use the Wald method [28] and model comparison uses the “D1” method for multivariate Wald tests [30].

## Results

### Stressors during lockdown

None of the participants reported becoming infected with COVID-19 during the study. This is consistent with the low rates of COVID in this province at the time. During the early lockdown (T3) stressful events scale total scores were on average 32.5 (SD 12.7). This corresponds to an average item rating of 2.02 (SD = 0.79) on the 1–5 scale. The most commonly identified stressful items in the questionnaire (i.e. rated severe or very severe) were: the increasing daily death toll (25.3%), lack of supplies of personal protective equipment (PPE; 21.2%), increasing daily incidence (20.2%), school closures (19.0%), medical staff infections and risk of hospitals being overwhelmed (17.0%), confirmed cases in your area (15.5%), and learning online rather than face-to-face (14.0%). As expected, we observed systematic reductions in the total stress scores from T3 to T4, as the lockdown eased: average change in the total score was − 2.17 (SE = 0.39; *t*_(391.88)_ = − 5.51, *p* < 0.0001, Cohen’s *d*: 0.179). At an item level, the proportion endorsing severe or very severe stress decreased for all but one item. Largest decreases were seen for lack of supplies of PPE (− 7.44%, McNemar *χ*^2^ = 23.7, *p* < 0.0001), news about the virus (− 5.26%, McNemar *χ*^2^ = 22.0, *p* < 0.0001), daily death toll (− 6.29%, McNemar *χ*^2^ = 14.1, *p* < 0.0002), school closures (− 5.26%, McNemar *χ*^2^ = 13.5, *p* < 0.0003). The only item to show an increase was stress about the number of confirmed cases in your area which was endorsed by an additional 1.37%, however, this was not a statistically significant change (McNemar *χ*^2^ = 0.776, *p* = 0.378). Further detail is available in Supplementary Table 2.

### The mental health impact of the COVID-19 lockdown

**Fig. 2:**
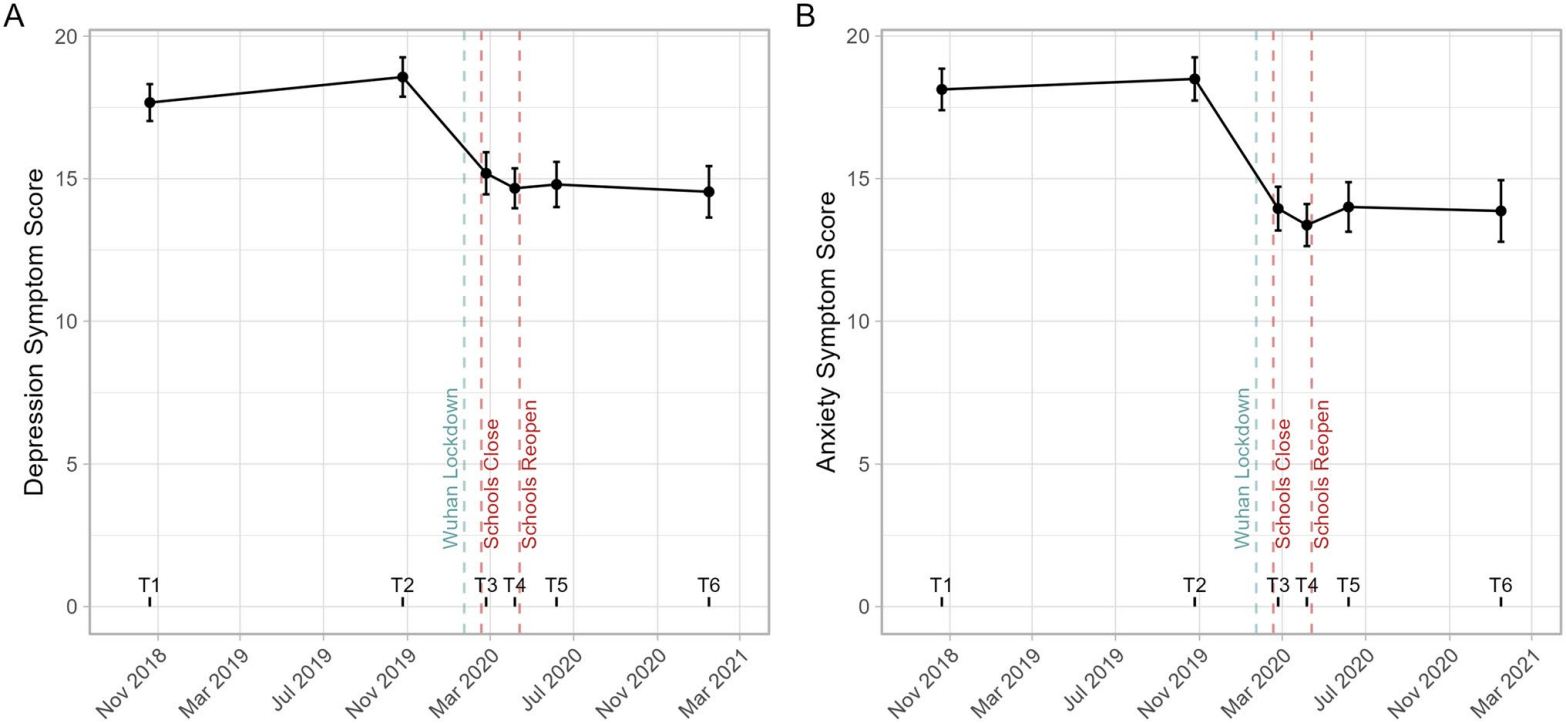
Depression and anxiety over time. Mean symptom scores for Depression (the Child Mood and Feelings Questionnaire; **A** Left) and Anxiety (the Screen for Child Anxiety Related Emotional Disorders; **B** Right). Error bars display 95% confidence intervals for the mean. The *x*-axis presents the time course of the study, with labels marking the observed timepoints (T1–T6). Vertical reference lines highlight key dates for context. The dates of school closure (2020-02-17) and reopening (2020-04-13) in Chenzhou are marked in red. The blue line indicates the first lockdown restrictions in China (2020-01-23, Wuhan, in neighbouring Hubei province), this coincided with the lunar new year holiday meaning student[28]s in Chenzhou were last in school prior to this date. Missing observations were imputed, and results are pooled over multiple imputations using Rubin’s rules [28]. For statistical comparisons between timepoints see Table 1

Self-report total symptom scores for depression (MFQ) and anxiety (SCARED) are plotted for each timepoint in Fig. 2, and pairwise comparisons are presented in Table 1.

**Table 1:** Pairwise comparisons of mental health scores

Scale/timepoint	Estimate	Std error	Cohen’s *d*	*df*	*T* statistic	*p* value
MFQ						
T2–T1	0.90	0.22	0.1040	880.9	4.07	< 0.001
T3–T1	− 2.48	0.36	− 0.1772	601.4	− 6.94	< 0.0001
T3–T2	− 3.38	0.34	− 0.2501	571.8	− 9.80	< 0.0001
T4–T1	− 3.01	0.34	− 0.2254	1053.2	− 8.83	< 0.0001
T4–T2	− 3.90	0.34	− 0.2938	1033.9	− 11.51	< 0.0001
T4–T3	− 0.53	0.30	− 0.0452	413.3	− 1.77	N.S
T5–T1	− 2.87	0.39	− 0.1883	572.5	− 7.38	< 0.0001
T5–T2	− 3.77	0.40	− 0.2435	672.7	− 9.54	< 0.0001
T5–T3	− 0.39	0.37	− 0.0271	383.4	− 1.06	N.S
T5–T4	0.13	0.33	0.0105	596.1	0.41	N.S
T6–T1	− 3.13	0.46	− 0.1738	329.7	− 6.81	< 0.0001
T6–T2	− 4.03	0.46	− 0.2242	353.3	− 8.78	< 0.0001
T6–T3	− 0.65	0.44	− 0.0376	244.3	− 1.47	N.S
T6–T4	− 0.12	0.40	− 0.0078	284.5	− 0.31	N.S
T6–T5	− 0.26	0.42	− 0.0155	235.7	− 0.61	N.S
SCARED						
T2–T1	0.37	0.36	0.0259	844.7	1.02	N.S
T3–T1	− 4.18	0.44	− 0.2434	711.0	− 9.53	< 0.0001
T3–T2	− 4.55	0.38	− 0.3041	506.2	− 11.91	< 0.0001
T4–T1	− 4.76	0.42	− 0.2900	1187.9	− 11.36	< 0.0001
T4–T2	− 5.13	0.37	− 0.3505	982.7	− 13.73	< 0.0001
T4–T3	− 0.58	0.31	− 0.0478	436.0	− 1.87	N.S
T5–T1	− 4.12	0.47	− 0.2220	730.2	− 8.69	< 0.0001
T5–T2	− 4.49	0.44	− 0.2607	680.2	− 10.21	< 0.0001
T5–T3	0.06	0.40	0.0037	376.5	0.15	N.S
T5–T4	0.64	0.35	0.0467	639.6	1.83	N.S
T6–T1	− 4.26	0.57	− 0.1911	323.5	− 7.49	< 0.0001
T6–T2	− 4.63	0.55	− 0.2137	256.5	− 8.37	< 0.0001
T6–T3	− 0.08	0.53	− 0.0040	213.0	− 0.16	N.S
T6–T4	0.49	0.48	0.0261	209.9	1.02	N.S
T6–T5	− 0.14	0.51	− 0.0070	211.9	− 0.27	N.S

Fixed effects estimates pooled over 100 multiple imputations of missing data using Rubin’s rules and associated Wald-tests. Degrees of freedom (*df*) are adjusted for missing information and the relative increase in variance due to imputation using the Barnard & Rubin equation. Estimates and standard errors are in the units of the questionnaire total scores. *MFQ* Child Mood and Feelings Questionnaire, *SCARED* Screen for Child Anxiety Related Emotional Disorders.

For the key question of a lockdown effect, we found both depression and anxiety total scores decreased significantly following lockdown (i.e. at time point T3): depression T3–T2 = − 3.37 (SE = 0.345), *T*_(572)_ = − 9.80, *p* < 0.0001, Cohen’s *D* = − 0.25; anxiety T3–T2 = − 4.55 (SE = 0.382), *T*_(506)_ = − 11.9, *p* < 0.0001, Cohen’s *D* = − 0.30. These T3 decreases were also significant relative to the previous year’s assessment (T1; see Table 1). The same pattern of statistically significant decreases from T2 and T1 observations was seen over all subsequent time points to T3 (pairwise comparisons presented in Table 1). Comparable results were obtained without using imputation by employing a pairwise complete-cases analysis (Supplementary Table S3).

After the immediate impact of the lockdown at T3 there were no subsequent significant differences in anxiety or depression scores with time, either as pairwise differences between time points (see Table 1) or as a linear trend with time: depression annualised trend estimate: − 0.516 (SE = 0.472), *T*(236) = − 1.09, *p* = 0.275, Cohen’s *D* = 0.028; anxiety annualised trend estimate: 0.148 (SE = 0.580), *T*(195) = 0.255, *p* = 0.80, Cohen’s *D* = 0.006.

### Predictors of mental health outcomes

Models to evaluate predictors of the change in symptom scores between T2 and T3 (adjusted for T2 scores) are summarised in Table 2. They reveal significant linear effects of the Covid Stressful Events scale total score such that each additional point was associated with greater scores (i.e. a lower reduction) in depression (+ 0.11, SE = 0.026, *p* < 0.0001) and anxiety (+ 0.11, SE = 0.036, *p* < 0.0001). Age and gender were not significant predictors of change and no predictors interacted significantly with the baseline scale score. We next probed estimated marginal means from these models to estimate the cross-over point where the effect of covid stress would start to reverse the secular trend. This revealed participants with CSE scores of 64 and 77 would show on average no reduction in depression and anxiety, respectively. These are extreme values in this sample (98.9th and 99.6th percentiles, respectively) and suggest that although self-reported covid stress was associated with a lower level of lockdown-related improvement in scores, it was a relatively small effect.

**Table 2:** Predictors of change from T2 to T3

Scale/model term	*b*	std error	statistic	*df*	*p* value
Depression (MFQ)					
T2 Scale Score	− 0.441	0.044	− 10.078	548.7	< 0.0001*
Age	0.428	0.467	0.917	518.4	0.360
Gender:F	− 0.835	0.697	− 1.197	464.9	0.232
CSE Score	0.108	0.026	4.173	430.2	< 0.0001*
Age × T2 Scale Score	− 0.054	0.033	− 1.629	692.5	0.104
Gender:F × T2 Scale Score	0.073	0.051	1.432	597.6	0.153
CSE Score × T2 Scale Score	0.001	0.002	0.675	557.4	0.500
Anxiety (SCARED)					
T2 Scale Score	− 0.496	0.044	− 11.261	480.4	< 0.0001*
Age	0.589	0.475	1.240	646.6	0.216
Gender:F	0.541	0.717	0.755	566.4	0.451
CSE Score	0.111	0.027	4.034	423.5	< 0.0001*
Age × T2 Scale Score	− 0.047	0.036	− 1.313	595.7	0.190
Gender:F × T2 Scale Score	0.078	0.053	1.459	434.8	0.145
CSE Score × T2 Scale Score	0.003	0.002	1.520	318.0	0.130

Unstandardised multiple linear regression coefficients (‘*b*’) from models predicting the lockdown-related change from T2–T3 in Depression (MFQ) and Anxiety (SCARED) total scores. In each case the model provided significant goodness-of-fit to the data by a D1 test—MFQ: *F*_(7,1248.4)_ = 40.52, *p* < 0.0001, R^2^ adjusted: 0.211; SCARED: *F*_(7,1209.3)_ = 49.01, *p* < 0.0001, *R*^2^ adjusted: 0.253

## Discussion

This study highlighted both the impact during the start and ongoing nature of the pandemic, and the potentially long-term impact of the pandemic on adolescent mental health compared with prior to the 2020 Chinese lockdown. Contrary to common expectations, we found a substantial decrease in depressive and anxiety symptoms compared with the previous waves, suggesting that mental health levels in Chinese adolescents aged 14–17 years saw improvements with the lockdown. In 8 weeks after schools reopening, anxiety and depression were still significantly lower than before the lockdown. The improvement was even sustained a year after the lockdown, suggesting the positive effects were relatively long-lasting.

These findings were consistent with some previous studies [6, 7], which suggested that there was a positive impact of lockdown on adolescent mental health and wellbeing in China. Li et al. [7] investigated how mental health developed across before, during, and after the pandemic breakout and found that depression and insomnia were the highest before the pandemic, then decreased during home confinement, and continued to decline after the lockdown. Qu et al. [6] reported also that the incidence of anxiety and depression in 10,216 adolescents were lower after two months of home confinement than that before lockdown. They described this positive pattern could be due to the lower academic pressure after school closure. Indeed, a recent systematic review of longitudinal adolescent studies revealed decreases in anxiety and depression over the initial months of the first lockdown [8]. The authors speculated that there had been considerable resilience in mental health. Adolescents with pre-existing mental health conditions also appeared not to be affected by pandemic-related changes and uncertainties [11, 12], they reasoned that this unexpected result might be attributable to some sense of relaxation and shared cohesiveness. In sum, these findings did not support the expectation that adolescents would be negatively affected by pandemic-related changes and school closures.

However, our findings were inconsistent with some other longitudinal studies that have suggested child mental health worsened due to the negative effects of social deprivation caused by the lockdowns [31, 32]. These longitudinal studies, however, have covered rather short intervals, e.g., several weeks or a few months, or lacked pre-pandemic comparative baseline data [33]. They were further unable to determine whether the increase in symptoms was transient or persistent. Thus, we would argue that limited conclusions can be drawn from these studies.

Whether our results can be generalised to other countries requires further considerations. School-related problems are a major stressor that contributes to students’ mental health problems across the globe [34]. It has been suggested that Chinese adolescents place particular importance on education, and devote their energy and time to obtaining high academic performance to increase future development opportunities [35]. Compared with adolescents from Western countries, Chinese students endure greater academic stress from heavy homework burden, high competitive pressure, and high parents’ expectation [36]. Such academic stress and workload demands were significant predictors of school burnout and emotional issues [37]. Bullying is also common among Chinese school students. In 2019, over half (57.29%) of students said they had been bullied at school in the past one year, compared to 26.1% in 2016 (i.e. an increase of 31%) [38]. The school disruption of the COVID-19 lockdown may have provided unexpected benefits to escape these negative stressors resulting in a better sense of well-being. For example, students staying at home could experience alleviation from the strict school environment, with reduced academic stress, peer- and teacher-related pressures, more time to think, better quality of sleep and relaxation [39]. They further had more opportunities for play-related activity and rest, as well as more independence and freedom to expand their autonomy [40]. These changes are likely to have contributed to reducing psychological distress and improving well-being, provided that their family and home environment supported these positive changes.

This explanation would be consistent with the finding that 80% of Chinese children and adolescents in primary and secondary school were satisfied with their life status during lockdown, and 21.4% of participants became more satisfied with life than before the pandemic [41]. Chinese adolescents also experienced strong positive changes in multiple life outcomes (e.g. relationships, physical activity, sleep, work) over the national lockdown period [42]. Outside of China, there were other studies pointing in the same direction: An overall reduction in anxiety and an increase in wellbeing were found in students aged 13–14 during the lockdown in England [43], and 90% of parents reported improvements in their children’s mental health compared with before the COVID-19 period in India [44]. In Canada, almost half of adolescents reported that the pandemic exerted positive effects, with more time to spend with one’s family[45], and more time to sleep, as well as increased psychosomatic health [46, 47]. These findings suggested that school closures may have protected pupils from some of the usual factors which can lead to poor mental health, including academic pressures, school bullying, and more subtle challenges in negotiating relationships with peers and teachers.

In contrast, other studies have argued that being confined to one’s home could severely limit an adolescent’s capacity for in-person social interaction and so harm their mental health [48]. However, some data suggested that despite adhering to the physical distancing measures, some people were still able to maintain pre-pandemic levels of social connection [49]. The decrease in adolescents’ face-to-face contact might have been less detrimental due to widespread access to digital forms of social interaction through social media [50]. Moreover, evidence has already emerged of a positive impact of social media on teenagers during COVID-19 in China [51]. Online contacts via social media had been quite helpful to deal with loneliness, boredom and anxiety as a constructive coping strategy for adolescents [52], and to maintain their social connections including to stay in touch with friends and schools during COVID-19 lockdowns [53].

Despite the substantial negative effects of lockdown on everyday life, a resilience perspective suggested improved family closeness during this stressful time [54]. Indeed, in another study, the majority of parents reported the lockdown improved their relationship with their children including engaging in more everyday activities with their children [55], showing more physical affection, warmth and love toward their children [56]. In our previous study, Chinese children and adolescents reported a significant decrease in different types of child maltreatment during lockdown [4]. These potentially beneficial effects may also explain why positive psychological functioning such as wellbeing, life satisfaction, or connectedness were unaffected by the COVID-19 lockdown in the general population [1].

On a more cautionary note, several limitations of our study need to be considered. First, we note limitations in the scales employed. All were self-report, and although the SCARED and MFQ-C are well established, the Covid Stressful Events scale was developed rapidly in response to the pandemic and so lacks validation. However, we note that this scale’s reliability (Cronbach’s Alpha) was excellent in this sample. Further, scales assessing other potential causes of mental health improvements (i.e. assessing the school and family environment) were not obtained, and so there is a need for future research to understand the potential mechanisms. Second, although the sample was large, it was a convenience sample taken from one high school which limits the generalizability of the results. Although the sex ratio of the school year groups was not recorded at the time, we presume this population was balanced and so our 70% female sample reflects a differing participation rate in research between males and females. Another potential limitation was the incomplete follow-up. Missingness was especially present for the final observation (T6) when older students had graduated, and to a lesser degree at T3 when many students were taking part in the Chinese Spring Festival holiday and so were away from school in their rural hometowns. Furthermore, some participants may have been unable or unwilling to complete the online survey when we transitioned from paper-and-pencil to an online format, this appears to be a limited effect as 97.7% completed at least one online survey. Also, our analysis focused on the average response to the pandemic and identifying distinct trajectories of adjustment using latent growth mixture modelling should be examined in future studies, because it is unclear how people with previous more severe mental health conditions, such as major depressive disorder, were affected by the pandemic. Finally, our findings, drawn from a single country, may not be generalizable to adolescents in other regions of the world. Different countries have widely varied in their response to COVID-19 so some of the strategies undertaken in China, and how this affected adolescent mental health, will not be relevant for other countries. However, it is hoped that useful findings can be taken from the effect of the wider remit of restrictions that many countries have introduced on adolescents’ mental health.

## Conclusion

To our knowledge, this is one of the first longitudinal studies on the adolescent mental health impact of COVID-19 covering almost all pandemic stages so far, with rather large intervals between the assessment waves. There was a significant short- and long-term improvement in depression and anxiety symptom during the national lockdown. We speculate that the benefits of lockdown on affective symptoms in adolescents in Hunan province were driven by relieving school-related stress or increasing resilience factors.

### Supplementary Information

Below is the link to the electronic supplementary material.

Supplementary file1 (PDF 252 KB)

## Data Availability

Anonymous data will be made available following the end of the pandemic.
