# Supplementary material for: Positive effects of the COVID-19 pandemic on depression and anxiety in Chinese adolescents
Source: Eur Child Adolesc Psychiatry. 2023 Jul 24;33(5):1551–61. doi: 10.1007/s00787-023-02263-z (PMC11098867; doi:10.1007/s00787-023-02263-z)
Supplement: Supplementary file 1 — Supplementary file1 (PDF 252 KB) [file 787_2023_2263_MOESM1_ESM.pdf]

Supplementary Information

Scale Measurement Invariance & Stressors During Lockdown

Article Title: Positive Effects of the COVID-19 Pandemic on depression and anxiety in Chinese adolescents
Journal Name: European Child & Adolescent Psychiatry
Authors: Xinhua Yang, Andrew J Lawrence, Phillippa Harrison, Yanlong Liu, Liangliang Chen, Chenglei Wang, Chao Yan, and Roland Zahn
Correspondence: Xinhua Yang,
Changning Mental Health Center,
299 Xiehe Road,
Changning District,
Shanghai, 200335
Tel.: +86 21 5219 2911
Email: wolaiye1974@163.com

Scale Measurement Invariance

Rationale & Methods

When comparing scores from psychometric instruments, researchers should consider whether the underlying constructs are being measured by the instrument items in a consistent way. In other words, for a longitudinal study: are the relationships between the construct and its indicator items similar between timepoints? This property can be assessed through analysis of measurement invariance [1].

To investigate measurement invariance in our data we performed tests over six time-points for the MFQ and SCARED instruments. Structural equation modelling (SEM) software (lavaan package v0.6.9 [2] for R software v4.1.2 [3]) was used with the semTools package [4] (v0.5-5.914) employed to construct syntax for configural (unrestricted) and scalar (thresholds and loadings constrained over time-points) invariance models. This process followed the recommendation and illustration provided by Svetina & colleagues [5]. Each scale was assessed with simple unifactorial Confirmatory Factor Analysis (CFA) model: Depression (33 ordinal

3-category indicator items from the MFQ) and Anxiety (41 ordinal 3-category indicator items from the SCARED). As all scale items had three response categories, the fixed-thresholds model was statistically equivalent to the configural model and so omitted [6]. Measurement invariance was judged by the comparison of nested models using the robust scaled Chi-squared difference test [7]. A significant difference (alpha = 0.05) between models was taken as evidence of a lack of measurement invariance between timepoints.

Results

The robust difference tests for nested WLSMV models were non-significant for both scales (MFQ scalar − configural: $\Delta\chi^2_{(160)}=134.56$, p=0.92; SCARED scalar − configural: $\Delta\chi^2_{(160)}=186.53$, p=0.07). This indicates an absence of substantial deviation from measurement invariance, thus scale totals can be directly compared and differences in total scores interpreted as reflecting differences in the constructs under investigation.

Supplementary Table S1 shows model statistics and fit measures. Although all models significantly deviated from perfect fit ($p<0.0001$), this was expected given the sample size [8]. Scaled model fit indices indicated acceptable fit [9] ($0.06 < RMSEA < 0.07$; CFI & TLI > 0.95 for MFQ and >0.935 for SCARED). We note that the comparison and interpretation of fit measures for categorical SEM using the appropriate mean and variance adjusted Weighted Least Squares (WLSMV) estimation method is not trivial (for example see Xia & Yang [10], and Kite et al [11]), and so we rely primarily on the robust chi-square difference test [7, 11] as reported above.

Supplementary Table S1: Measurement Invariance Model Fit Measures

**Outcome**	**Parameter**	**Unscaled**		**Scaled (Robust)**	
		configural	*scalar*	*configural*	*scalar*
**MFQ**	χ^2	15714.6	16056.6	18592.8	18467.0
	df	2970	3130	2970	3130
	p-value	<0.0001	<0.0001	<0.0001	<0.0001
	RMSEA	0.06245	0.06126	0.06914	0.06673
	CFI	0.99455	0.99447	0.96069	0.96141
	TLI	0.99419	0.99441	0.95807	0.96094
**SCARED**	χ^2	30429.4	30829.1	28754.7	28445.7
	df	4674	4874	4674	4874
	p-value	<0.0001	<0.0001	<0.0001	<0.0001
	RMSEA	0.07101	0.06981	0.06866	0.06652
	CFI	0.99025	0.99018	0.93995	0.94122
	TLI	0.98974	0.99009	0.93679	0.94067

df = degrees of freedom; RMSEA = Root Mean Squared Error of Approximation; CFI = Comparative Fit Index; TLI = Tucker-Lewis Index.

Supplementary Table S2: Stressors During Lockdown

Covid Questionnaire Item	T3: Early Lockdown					T4: Lockdown easing				
	None	Mild	Moderate	Severe	Very Severe	None	Mild	Moderate	Severe	Very Severe
1. restrictions on the free movement of citizens	337 (38.6%)	317 (36.3%)	159 (18.2%)	44 (5.0%)	17 (1.9%)	500 (57.2%)	231 (26.4%)	102 (11.7%)	26 (3.0%)	15 (1.7%)
2. testing of temperature and wearing masks in public	510 (58.4%)	205 (23.5%)	109 (12.5%)	27 (3.1%)	23 (2.6%)	484 (55.4%)	250 (28.6%)	106 (12.1%)	21 (2.4%)	13 (1.5%)
3. closed residential community	476 (54.5%)	233 (26.7%)	108 (12.4%)	42 (4.8%)	15 (1.7%)	490 (56.1%)	249 (28.5%)	101 (11.6%)	21 (2.4%)	13 (1.5%)
4. being unable to meet friends and relatives	430 (49.2%)	247 (28.3%)	115 (13.2%)	46 (5.3%)	36 (4.1%)	447 (51.1%)	266 (30.4%)	107 (12.2%)	32 (3.7%)	22 (2.5%)
5. widespread news and information about virus	503 (57.6%)	192 (22.0%)	90 (10.3%)	57 (6.5%)	32 (3.7%)	493 (56.4%)	241 (27.6%)	97 (11.1%)	29 (3.3%)	14 (1.6%)
6. mandatory reported health situation everyday	519 (59.4%)	216 (24.7%)	77 (8.8%)	40 (4.6%)	22 (2.5%)	492 (56.3%)	240 (27.5%)	107 (12.2%)	17 (1.9%)	18 (2.1%)
7. being unable to exercise outdoors	357 (40.8%)	280 (32.0%)	141 (16.1%)	53 (6.1%)	43 (4.9%)	431 (49.3%)	243 (27.8%)	130 (14.9%)	39 (4.5%)	31 (3.5%)
8. school closures	280 (32.0%)	225 (25.7%)	206 (23.6%)	86 (9.8%)	77 (8.8%)	347 (39.7%)	237 (27.1%)	173 (19.8%)	71 (8.1%)	46 (5.3%)
9. learning online rather than face-to-face	351 (40.2%)	234 (26.8%)	161 (18.4%)	52 (5.9%)	76 (8.7%)	359 (41.1%)	259 (29.6%)	151 (17.3%)	54 (6.2%)	51 (5.8%)
10. parents' management of children's learning	538 (61.6%)	198 (22.7%)	84 (9.6%)	33 (3.8%)	21 (2.4%)	491 (56.2%)	239 (27.3%)	101 (11.6%)	19 (2.2%)	24 (2.7%)
11. family fear of COVID-19	401 (45.9%)	286 (32.7%)	122 (14.0%)	44 (5.0%)	21 (2.4%)	431 (49.3%)	274 (31.4%)	118 (13.5%)	32 (3.7%)	19 (2.2%)
12. hospitals being overwhelmed	293 (33.5%)	264 (30.2%)	171 (19.6%)	88 (10.1%)	58 (6.6%)	343 (39.2%)	244 (27.9%)	171 (19.6%)	65 (7.4%)	51 (5.8%)
13. lack of supplies of personal protective equipment	235 (26.9%)	260 (29.7%)	193 (22.1%)	110 (12.6%)	76 (8.7%)	332 (38.0%)	254 (29.1%)	167 (19.1%)	75 (8.6%)	46 (5.3%)
14. confirmed cases in your area	426 (48.7%)	187 (21.4%)	131 (15.0%)	79 (9.0%)	51 (5.8%)	382 (43.7%)	226 (25.9%)	124 (14.2%)	79 (9.0%)	63 (7.2%)
15. increasing daily incidence	257 (29.4%)	237 (27.1%)	207 (23.7%)	94 (10.8%)	79 (9.0%)	336 (38.4%)	221 (25.3%)	174 (19.9%)	84 (9.6%)	59 (6.8%)
16. increasing daily deaths toll	227 (26.0%)	227 (26.0%)	200 (22.9%)	116 (13.3%)	104 (11.9%)	318 (36.4%)	208 (23.8%)	183 (20.9%)	88 (10.1%)	77 (8.8%)

Supplementary Table S2 shows the number and percentage at timepoints T3 and T4 endorsing the 5-point likert responses for the 16 items in the Covid Questionnaire.

Supplementary Table S3: Pairwise Comparisons of Mental Health Scores

Scale/ Timepoint	Estimate	Std Error	Cohen's D	df	T-statistic	p-value
**MFQ**						
**T2-T1**	0.83	0.23	0.109	1123	3.64	<0.001
**T3-T1**	-3.10	0.42	-0.257	822	-7.37	<0.0001
**T3-T2**	-3.74	0.40	-0.330	792	-9.30	<0.0001
**T4-T1**	-2.97	0.34	-0.247	1211	-8.61	<0.0001
**T4-T2**	-3.67	0.36	-0.302	1158	-10.28	<0.0001
**T4-T3**	-0.78	0.31	-0.084	873	-2.48	0.013
**T5-T1**	-2.72	0.42	-0.215	912	-6.50	<0.0001
**T5-T2**	-4.21	0.45	-0.313	872	-9.26	<0.0001
**T5-T3**	-0.41	0.42	-0.037	715	-0.99	N.S.
**T5-T4**	-0.02	0.34	-0.002	1033	-0.05	N.S.
**T6-T1**	-2.15	0.57	-0.167	507	-3.77	<0.001
**T6-T2**	-3.76	0.57	-0.283	547	-6.63	<0.0001
**T6-T3**	-0.48	0.57	-0.043	400	-0.86	N.S.
**T6-T4**	0.02	0.46	0.002	604	0.04	N.S.
**T6-T5**	-0.41	0.50	-0.036	527	-0.82	N.S.
**SCARED**						
**T2-T1**	0.27	0.39	0.021	1092	0.69	N.S.
**T3-T1**	-4.24	0.52	-0.286	807	-8.14	<0.0001
**T3-T2**	-4.76	0.44	-0.392	778	-10.94	<0.0001
**T4-T1**	-4.99	0.44	-0.330	1193	-11.39	<0.0001
**T4-T2**	-5.19	0.39	-0.394	1135	-13.29	<0.0001
**T4-T3**	-0.58	0.32	-0.061	873	-1.79	N.S.
**T5-T1**	-4.24	0.54	-0.261	904	-7.84	<0.0001
**T5-T2**	-5.11	0.51	-0.343	860	-10.06	<0.0001
**T5-T3**	-0.06	0.45	-0.005	715	-0.14	N.S.
**T5-T4**	0.47	0.37	0.040	1033	1.28	N.S.
**T6-T1**	-3.33	0.77	-0.193	503	-4.33	<0.0001
**T6-T2**	-5.06	0.65	-0.337	531	-7.78	<0.0001
**T6-T3**	0.35	0.66	0.026	400	0.53	N.S.
**T6-T4**	0.45	0.49	0.037	604	0.92	N.S.
**T6-T5**	-0.06	0.58	-0.005	527	-0.10	N.S.

Supplementary Table S3 presents fixed effects estimates for change between timepoints using pairwise complete data. Estimates and standard errors are in the units of the questionnaire total scores.

df = degrees of freedom, MFQ = Child Mood and Feelings Questionnaire, SCARED = Screen for Child Anxiety Related Emotional Disorders.

Citations

1. Putnick DL, Bornstein MH (2016) Measurement Invariance Conventions and Reporting: The State of the Art and Future Directions for Psychological Research. Dev Rev 41:71–90. https://doi.org/10.1016/j.dr.2016.06.004

2. Rosseel Y (2012) lavaan: An R Package for Structural Equation Modeling. Journal of Statistical Software 48:1–36. https://doi.org/10.18637/jss.v048.i02

3. R Core Team (2021) R: A Language and Environment for Statistical Computing. R Foundation for Statistical Computing, Vienna, Austria

4. Jorgensen TD, Pornprasertmanit S, Schoemann AM, Rosseel Y (2021) semTools: Useful tools for structural equation modeling

5. Svetina D, Rutkowski L, Rutkowski D (2020) Multiple-Group Invariance with Categorical Outcomes Using Updated Guidelines: An Illustration Using Mplus and the lavaan/semTools Packages. Structural Equation Modeling: A Multidisciplinary Journal 27:111–130. https://doi.org/10.1080/10705511.2019.1602776

6. Wu H, Estabrook R (2016) Identification of Confirmatory Factor Analysis Models of Different Levels of Invariance for Ordered Categorical Outcomes. Psychometrika 81:1014–1045. https://doi.org/10.1007/s11336-016-9506-0

7. Satorra A (2000) Scaled and Adjusted Restricted Tests in Multi-Sample Analysis of Moment Structures. In: Heijmans RDH, Pollock DSG, Satorra A (eds) Innovations in Multivariate Statistical Analysis: A Festschrift for Heinz Neudecker. Springer US, Boston, MA, pp 233–247

8. Bentler PM, Bonett DG (1980) Significance tests and goodness of fit in the analysis of covariance structures. Psychological Bulletin 88:588–606. https://doi.org/10.1037/0033-2909.88.3.588

9. Hu L, Bentler PM (1999) Cutoff criteria for fit indexes in covariance structure analysis: Conventional criteria versus new alternatives. Structural Equation Modeling: A Multidisciplinary Journal 6:1–55. https://doi.org/10.1080/10705519909540118

10. Xia Y, Yang Y (2019) RMSEA, CFI, and TLI in structural equation modeling with ordered categorical data: The story they tell depends on the estimation methods. Behav Res 51:409–428. https://doi.org/10.3758/s13428-018-1055-2

11. Kite BA, Jorgensen TD, Chen P-Y (2018) Random Permutation Testing Applied to Measurement Invariance Testing with Ordered-Categorical Indicators. Structural Equation Modeling: A Multidisciplinary Journal 25:573–587. https://doi.org/10.1080/10705511.2017.1421467
